# Perceptions, maltreatment and religion as predictors of the psycho-emotional impact on nurses during the COVID-19 pandemic

**DOI:** 10.1590/0034-7167-2022-0768

**Published:** 2023-08-21

**Authors:** Jhon Alex Zeladita-Huaman, Stefanny Lizbeth De la Cruz-Espinoza, Gabriela Samillán-Yncio, Rosa Castro-Murillo, Eduardo Franco-Chalco, Roberto Zegarra-Chapoñan

**Affiliations:** I Universidad Nacional Mayor de San Marcos. Lima, Lima, Perú; II Hospital Hermilio Valdizán. Santa Anita, Lima, Perú.; III Universidad María Auxiliadora. San Juan de Lurigancho, Lima, Perú.

**Keywords:** Stress Psychological, Anxiety, Depression, Mental Health, Perception, Estresse Psicológico, Ansiedade, Depressão, Saúde Mental, Percepção, Estrés Psicológico, Ansiedad, Depresión, Salud Mental, Percepción

## Abstract

**Objectives:**

to analyze the relationship between perceptions, abuse and religion with the psycho-emotional impact on nurses during the COVID-19 pandemic.

**Methods:**

descriptive-analytical cross-sectional study. It took place between 2020 and 2021 and a total of 319 clinical nurses in Peru were interviewed using the DASS-21. Associations were assessed using Spearman’s Rho and multiple regression.

**Results:**

18.5% had some degree of stress; 50.2%, anxiety and 29.1%, depression. Experience of abuse, self-perception of mental health and religion were predictors of stress, anxiety and depression. The length of work experience predicts stress and anxiety. In addition, self-perception of information and gender were predictors of depression.

**Conclusions:**

peruvian nurses have high levels of stress, anxiety and depression, and this psycho-emotional impact was associated with perceptions, experiences of abuse and religion.

## RESUMEN

**Objetivos:**

analizar la relación entre percepciones, maltrato y religión con el impacto psicoemocional en enfermeros durante la pandemia por covid-19.

**Métodos:**

estudio transversal descriptivo-analítico. Fue realizado entre los años 2020 y 2021; se encuestó a 319 enfermeros asistenciales de Perú mediante DASS-21; se evaluaron asociaciones mediante Rho de Spearman y regresión múltiple.

**Resultados:**

el 18,5 % presentó algún grado de estrés; el 50,2 %, ansiedad y el 29,1 %, depresión. La experiencia de maltrato, la autopercepción de la salud mental y la religión fueron predictores de estrés, ansiedad y depresión. El tiempo de experiencia laboral predice el estrés y ansiedad. Además, la autopercepción de la información y el género fueron predictores de la depresión.

**Conclusiones:**

los enfermeros peruanos presentaron altos niveles de estrés, ansiedad y depresión; y este impacto psicoemocional estuvo asociado a las percepciones, experiencias de maltrato y la religión.

## RESUMO

**Objetivos:**

analisar a relação entre percepções, abuso e religião com o impacto psicoemocional nos enfermeiros durante a pandemia de COVID-19.

**Métodos:**

estudo transversal descritivo-analítico. Realizado entre 2020 e 2021; foram entrevistados 319 enfermeiros assistenciais no Peru por meio do DASS-21; as associações foram avaliadas usando Rho de Spearman e regressão múltipla.

**Resultados:**

18,5% apresentaram algum grau de estresse; 50,2%, ansiedade e 29,1%, depressão. Experiência de abuso, autopercepção de saúde mental e religião foram preditores de estresse, ansiedade e depressão. O tempo de experiência de trabalho prediz estresse e ansiedade. Além disso, a autopercepção das informações e o gênero foram preditores de depressão.

**Conclusões:**

os enfermeiros peruanos apresentam altos níveis de estresse, ansiedade e depressão; e esse impacto psicoemocional foi associado a percepções, experiências de abuso e religião.

## INTRODUCTION

COVID-19 has changed daily life and professional life. Despite the various containment measures adopted worldwide, the growing number of infected people has caused the collapse of health services worldwide^(1)^. This situation highlighted the important role of nurses, who from their work on the front lines have faced pain, situational crises, death and various ethical dilemmas, in addition to the scarcity of material resources, protective equipment, human resources and workload. Consequently, these working conditions caused mental health problems in nurses due to the high physical, intellectual and emotional demands^(2)^.

One of the scales widely used to assess the impact of COVID-19 on the mental health of nurses was the Depression, Anxiety and Stress Scale (DASS-21)^(3, 4)^. Using this scale, studies carried out with nurses at the beginning of the pandemic highlight a high prevalence of emotional reactions such as depression (56.7-57.8%), anxiety (49.6-42.2%) and stress (50.1% )^(3, 4)^, while in the third post-pandemic year, a decrease in the prevalence of these mental health problems is reported^(5)^. Another study carried out with Brazilian nurses reports a prevalence of depression in 25% and anxiety in 48.9%^(6)^. Likewise, in The COVID-19 Health Care Workers Study (HEROES), carried out in 2020 in 26 countries, it was highlighted that approximately a quarter of health professionals manifested depressive symptoms, that is, about 15% considered committing suicide^(7)^. In Peru, 39.1% of nurses had anxiety, while 24.6% had depression and only 8.8% had stress^(8)^.

Scientific evidence indicates that when health professionals experience situations of violence, they can develop mental health problems^(9)^. In this regard, a study carried out with doctors points out that violence in the workplace was a predictor of stress^(2)^. In addition, in times of COVID-19, those who felt discriminated due to the outbreak^(10)^ and those who suffered psychological abuse showed greater stress, anxiety and depression. The few studies carried out with nurses highlighted that, when they are discriminated due to their work nature, they are at greater risk of suffering stress, anxiety and depression^(11)^. Receiving negative comments is associated with high scores of depression and anxiety^(12)^, while verbal violence and sexual harassment increased depressive symptoms, which increased the intention to change services^(13)^.

Researches carried out during the COVID-19 pandemic highlights that one of the protective factors for psycho-emotional problems is religion. In this regard, a study carried out in Singapore points out that the practice of religious activities, such as prayers and meditation, was associated with low levels of anxiety in patients with psychosis^(14)^. Similarly, another study conducted among people living in Malaysian revealed that both external and internal forms of religiosity are associated with lower levels of stress and that religiosity can also moderate stress derived from various perceptions about COVID-19^(15)^. On the other hand, a cohort study conducted with military personnel in the United States points to an association between religion and the risk of incidents of post-traumatic stress disorder, suicidal ideation and dangerous alcohol consumption^(16)^. Regarding health personnel, the HEROES study, which included eleven Latin American countries, found that considering oneself a religious person reduces risks, psychic suffering and depressive symptoms^(7)^. However, no studies were found that evaluated the association between these variables in nurses.

The perception of illness, according to the self-regulation model, is a construct that describes how an individual perceives the symptoms, development, chronicity, causes and consequences of their illness, through two mental processing pathways: cognitive and emotional^(17)^. In this regard, in Peru, a study reported that half of the nurses in the first level of care self-perceived their health as poor^(18)^. On the other hand, in Brazil, a study showed that the self-perception of a poor mental health state was the result of post-traumatic stress, anxiety and depression^(19)^.

Most studies that analyzed the factors associated with stress, anxiety and depression in nurses in times of COVID-19 coincided in pointing out their association with gender, age and having been diagnosed with this disease^(3, 4, 8, 20)^. However, there is discrepancy in the association of psycho-emotional impact with length of professional experience, while a study points to a direct relationship with stress^(21)^, anxiety and depression^(22)^, another study indicates an indirect correlation^(23)^ and another one indicates that there is no association^(5)^. In addition, there are few studies in Latin America that assess the association of abuse, religion and perceptions as predictors of the psycho-emotional impact on nurses.

## OBJECTIVES

To analyze the relationship between perceptions, abuse and religion with the psycho-emotional impact on nurses during the COVID-19 pandemic.

## METHODS

### Ethical aspects

The study was approved by a Research Ethics Committee. Prior to data collection, the Informed Consent Form was applied, virtually, to all participants.

### Design, study setting and period

Study with a quantitative approach, descriptive-analytical and cross-sectional level carried out in Lima-Peru between December 2020 and July 2021. The report was oriented according to the STROBE tool.

### Population and sample: inclusion and exclusion criteria

The sample consisted of nurses working in health facilities in Peru. The inclusion criteria were: having access to a device with internet access and being available to answer the questionnaire. Nurses living in other countries were excluded. Assuming a conservative measure that the emotional impact of COVID-19 will have a small effect on food consumption (f2 = 0.02) and considering a type I error probability equal to 0.05 and a minimum statistical power of 80%, the sample size is estimated at 311 individuals. Therefore, data collection was completed when a total of 319 participants responded. A non-probabilistic sampling was adopted for convenience.

### Study protocol

Through a virtual survey, using a self-administered Google Forms questionnaire, data collection was performed. Nurses were invited to participate in the study through the social networks of the health establishments and universities where they were studying the second specialization. To measure the psycho-emotional impact, the DASS-21 scale was used, validated in the Peruvian population^(24)^. This 21-item scale assesses depression, anxiety, and stress, which has been validated based on expert judgment. Likewise, through a pilot test, it was determined that it has high internal consistency, and it was determined by Cronbach’s Alpha for the depression subscale (0.854), anxiety subscale (0.837) and stress subscale (0.784).

In the same way, in the virtual form, the possible predictors were investigated: sociodemographic factors (gender, age, marital status, type of religion professed), labor factors (graduation, service in which they work, length of experience, number of establishments where they work), epidemiological factors such as having been diagnosed with COVID-19 at some point during the pandemic or whether any of their relatives have died. Self-perception of health, self-perception of mental health, perception of protection and availability of personal protective equipment, perception of information conveyed by the media and whether they had experiences of abuse by patients were also evaluated.

### Data analysis

At first, categorical variables were described using frequencies and percentages and continuous variables using mean, standard deviation and bivariate correlations. In this first descriptive analysis, it was possible to detect that two variables had missing cases. For this reason, data loss was found to be completely random (MCAR) with Little’s test. Likewise, it was identified that the variables stress, anxiety and depression do not follow a normal distribution. For further analysis of the study, these variables were transformed using Tukey’s power scale^(25)^, which allows the distribution of data to approach a distribution closer to normal. To work with missing data, the multiple imputation technique was used to generate 10 databases with complete data in order to adjust the regression models that responded to the objectives of this investigation. To identify the variables associated with stress, anxiety and depression, a multiple regression analysis was performed with variable selection by steps (stepwise). Since this analysis had to be carried out in each of the imputed databases, the recommendations of van Buuren^(26)^ were used, which indicate that the variables that are chosen more than 50% of the time and that contribute significantly for the AIC are included in the final model. Finally, once the regression model with the best predictive capacity was identified, the regression coefficients of the 10 databases were collapsed using Rubin’s rules^(27)^. The software R v4.2.1^(28)^ was used.

## RESULTS

A total of 319 nurses participated and 86.2% (272) of them were women. Regarding age, 43.9% (140) were between 34 and 40 years old, 27.3% (87) were under 34 years old and 28.8% (92) were over 40 years old. As for marital status, 53.3% (170) were single, 41.4% (132) were married or living with a partner, and 5.3% (17) were separated. As for religion, 76.5% (244) of the participants profess the Catholic religion, 14.1% (45) consider themselves evangelicals, 5.9% (19) claim to be agnostics and 3.5% (11) profess another religion. In addition, 45.5% (145) indicated that they were attending graduate school at the time of the survey. 43.4% (137) reported having suffered mistreatment by patients at the health unit where they worked. As for epidemiological characteristics, 49.2% (157) were diagnosed with COVID-19 at some point during the pandemic, while 41.4% (131) reported that a relative had COVID and 26.6% (85) that a close person died of COVID-19 (Table 1).

**Table 1:** Characteristics of surveyed nurses, Peru, 2020-2021

Variable	n	%
Health perception		
Better	67	21.0
Same	142	44.5
Worse	110	34.5
Mental health perception		
Good	144	45.1
Regular	161	50.5
Poor	14	4.4
Place where they work		
Primary Care	133	41.7
Others (hospitals, clinic or polyclinic)	186	58.3
Work experience (years)		
Less than 6	193	60.5
From 6 to 15	82	25.7
Over 15	44	13.8
Number of establishments where they work		
One	237	74.3
Two	82	25.7
Perception of protection from personal protective equipment		
Safe	147	62.8
Not so safe	82	35.1
Not safe	5	2.1
Availability of personal protective equipment		
Always	131	41.1
Sometimes	139	43.6
Almost never	49	15.4
Perception of media information		
Adequate	159	49.9
Indifferent	76	23.8
Inappropriate	84	26.3

It was found that 18.5% of nurses had some level of stress, while 50.2%, anxiety and 29.1%, depression. Regarding the correlations, it can be observed that the three variables present a positive and strong correlation with each other (0.67 > Rho’s < 0.703) (Table 2).

**Table 2:** Level, means, standard deviations and correlations between stress, anxiety and depression reported by nurses, Peru, 2020-2021

	Normal %	Mild %	Moderate %	Severe %	Very severe %	M(SD)	Spearman's Rho		
							1	2	3
1. Stress	81.5	10.4	5.6	2.2	0.3	5.1(3.1)	-		
2. Anxiety	49.8	9.5	24.1	6.9	9.7	4.3(3.6)	0.703*	-	
3. Depression	70.8	11.3	11.3	4.1	2.5	3.9(3.5)	0.703*	0.67*	-

* *p < 0.001; M – Mean, SD – Standard derivation.*

The multiple regression model for predicting stress levels (Table 3) specifically reports that those with the highest stress scores are people with more than 15 years of work experience, compared to those with less than 6 years (B = 0.19, p <0.001), people who perceive themselves as having fair mental health (B = 0.28, p <0.001) and those who perceive themselves as having poor mental health (B = 0.25, p <0.001). On the other hand, participants who reported no experiences of neglect or abuse reported lower levels of stress than people who reported those experiences (B = -0.10, p = 0.045). Finally, regarding religion, it is observed that Catholic participants reported lower levels of stress than Evangelical participants (B = 0.20, p <0.001) and agnostic participants (B = 0.23, p <0.001). This model explains 27% of the total stress variation.

**Table 3:** Multiple regression model with stepwise variable selection to predict nurses’ stress scores, Peru, 2020-2021

Variable	b*	EE^†^	B^‡^	p^§^
Intercept	2.59	0.22		<0.001
Work experience (6-15 years)	0.12	0.15	0.04	0.407
Professional experience (> 15 years)	0.69	0.18	0.19	<0.001
Perception of mental health (Regular)	0.70	0.13	0.28	<0.001
Perception of mental health (poor)	1.51	0.31	0.25	<0.001
Experience of abuse (no)	−0.25	0.13	−0.10	0.045
Religion (evangelical)	0.75	0.19	0.20	<0.001
Religion (agnostic)	1.25	0.28	0.23	<0.001
Religion (other)	0.00	0.34	0.00	0.992
Service where you work (others)	−0.14	0.13	−0.05	0.288
Gender (woman)	−0.28	0.20	−0.08	0.158
R2	0.27			

* *b – Non-standard coefficient; †SE – Standard error; ‡B – Standardized coefficient; §p – Statistical significance. Note: The reference category for the variable working time was "< 6 years", the reference category for the perception of mental health was "Good", the reference category for the experience of neglect or abuse was " Yes", the category for religion was "Catholic", the reference category for the service where they work was "Primary Care" and the reference category for gender was "Male".*

The multiple regression model for predicting anxiety levels (Table 4) reports that those with the highest anxiety scores were professionals with more than 15 years of professional experience (B = 0.10, p = 0.045) and those who perceived having a regular (B = 0.29, p <0.001) and poor (B = 0.29, p <0.001) mental health. On the other hand, the participants who reported having the lowest anxiety score were those who did not suffer abuse (B = -0.15, p = 0.002), those who profess the Catholic religion (B = 0.13, p = 0.008) and agnostics (B = 0.21, p < 0.001) compared with evangelicals. However, Catholic participants had higher levels of anxiety than participants of other religions (B = −0.10, p = 0.047). This model, in general, explains 25% of the total anxiety variance.

**Table 4:** Multiple regression model with stepwise variable selection to predict nurses’ anxiety scores, Peru, 2020-2021

Variable	b*	EE^†^	B^‡^	p^§^
Intercept	1.80	0.18		<0.001
Diagnosis of COVID-19 (no)	−0.20	0.13	−0.08	0.126
Work experience (6-15 years)	−0.10	0.15	−0.03	0.515
Professional experience (> 15 years)	0.39	0.19	0.10	0.042
Health perception (same)	−0.09	0.17	−0.03	0.602
Health perception (worse)	0.26	0.18	0.09	0.157
Perception of mental health (regular)	0.76	0.13	0.29	<0.001
Perception of mental health (poor)	1.83	0.32	0.29	<0.001
Experience of abuse (no)	−0.41	0.13	−0.15	0.002
Religion (evangelical)	0.51	0.19	0.13	0.008
Religion (agnostic)	1.14	0.27	0.21	<0.001
Religion (others)	−0.70	0.35	−0.10	0.047
R2	0.25			

* *b – Non-standard coefficient; †SE – Standard error; ‡B – Standardized coefficient; §p – Statistical significance. Note: The reference category for the variable diagnosis of COVID-19 was "Yes", the reference category for the variable working time was "< 6 years", the reference category for health perception was "Better", the category reference category for perceived mental health was “Good”, the reference category for experience of neglect or abuse was “Yes” and the reference category for religion was “Catholic”.*

Finally, the multiple regression model to predict levels of depression (Table 5) reports that people who perceived the highest depression score were men (B = −0.11, p = 0.036), who consider media information inappropriate (B = 0.12, p = 0.017), those who perceive their mental health to be regular (B = 0.34, p <0.001) and poor (B = 0.31, p <0.001). On the other hand, participants who reported the lowest depression score were those who had not experienced abuse (B = −0.11, p = 0.022), Catholic participants (B = 0.21, p < 0.001), and agnostics (B = 0.16, p = 0.001), compared with evangelical participants. This model explains 29% of the total variance of depression among nurses.

**Table 5:** Multiple regression model with stepwise variable selection to predict depression scores of nurses, Peru, 2020-2021

Variable	b*	EE^†^	B^‡^	p^§^
Intercept	1.69	0.17		<0.001
Perception of information (indifferent)	0.08	0.12	0.04	0.478
Perception of information (inadequate)	0.27	0.11	0.12	0.017
Perception of mental health (regular)	0.66	0.10	0.34	<0.001
Perception of mental health (poor)	1.48	0.23	0.31	<0.001
Experience of abuse (no)	−0.22	0.10	−0.11	0.022
Religion (evangelical)	0.61	0.14	0.21	<0.001
Religion (agnostic)	0.66	0.22	0.16	0.002
Religion (others)	0.03	0.26	0.00	0.921
Service (others)	−0.16	0.10	−0.08	0.101
Gender (woman)	−0.31	0.15	−0.11	0.036
R2	0.29			

* *b – Non-standard coefficient; †SE – Standard error; ‡B – Standardized coefficient; §p – Statistical significance. Note: The reference category for perception of information was "Adequate", the reference category for perception of mental health was "Good", the reference category for experience of neglect or abuse was "Yes", the reference category for religion was “Catholic”, the reference category for the service where he works was “Primary Care” and the reference category for gender was “Male”.*

## DISCUSSION

In this study carried out with Peruvian nurses, the main finding is that religion, self-perception of mental health and experiences of abuse by patients predict stress, anxiety and depression. Likewise, work experience predicts stress and anxiety and gender and perception of information about COVID-19 predict depression.

The association between having suffered abuse during care work with stress, anxiety and depression, found in this research, is consistent with studies carried out in China, where they highlight that nurses discriminated by the nature of their work and who have suffered violence at work^(11)^ and health professionals who have suffered violence during the pandemic^(9)^ are at greater risk of suffering these three psychological outcomes. It was reported that receiving negative comments is related to depression and anxiety^(12)^ and that discrimination^(10)^, violence at work and psychological abuse increase depressive symptoms^(13)^, stress and anxiety^(2, 19)^. Consequently, violence in the hospital work environment, as well as inadequate interpersonal relationships and discrimination, make health professionals perceive the work environment as unsafe, an aspect that would play a crucial role in the development of psycho-emotional problems^(11)^.

This problem becomes relevant because since the emergence of the pandemic, multiple cases of mistreatment of health professionals have been reported around the world, more frequently in South America^(29)^. Although in several countries like Peru laws have been enacted to guarantee the integrity of personnel working in health services^(30)^, it is not enough to control this problem. Therefore, to prevent health professionals from exposing themselves to situations that generate violence, it is necessary to develop public policies that guarantee safe environments in hospitals.

Another finding of this study was that people who profess the evangelical religion and agnostics (those who do not profess any religion) have higher scores of stress, anxiety and depression compared to those who consider themselves Catholics. This finding is in line with a study carried out in Ghana during the COVID-19 pandemic, in which they report that there are no differences between the percentage of residents who experience stress, anxiety and depression depending on the type of religion they profess (Christian, Islamic and agnostic)^(31)^. This difference is likely due to cultural differences between Latin American and African countries.

However, the association between religion and psycho-emotional impact reported in this study coincides with a study with a cultural focus carried out in Malaysia, where they found that there are differences between the type of religion professed and the perception of stress, and they specifically point out that Buddhists have higher stress scores than Christians and Muslims^(15)^. Furthermore, another study, using a regression model adjusted for gender and religion, found that negative religious coping (underlying spiritual tensions, spiritual discontent, and struggles with themselves, others, and the divine) was associated with stress, anxiety and depression^(14)^. Likewise, a report carried out in eleven countries in the Americas region highlights the consideration of a spiritual or religious person as a protective factor^(7)^. One explanation for this relationship could be that religious service is considered a protective factor for mental health because it provides a multidimensional experience that reserves time for reflection, in addition to favoring social support^(16)^.

In this research, it was found that nurses with more time on the job have higher scores in terms of stress and anxiety, but there are no differences in depression scores. Likewise, a study carried out with Chinese nurses working in pediatric services found that having more than 10 years of professional experience is a risk factor for stress^(21)^. In addition, another study carried out with Spanish nurses in which, through bivariate analysis, found that having more than 10 years of professional experience is associated with the risk of depression. However, the multivariate analysis reported that there is no association between these variables^(5)^. Similarly, another study carried out in Brazil, using bivariate analysis, points out differences between length of service in the profession (years) with stress and depression in nurses^(32)^.

It is important to highlight that there is a discrepancy in reporting the directionality of the relationship between length of work experience and psycho-emotional impact. While in Turkey^(22)^ and Peru^(8)^ they found that there is a direct relationship, in the United States they indicate that there is an inverse correlation^(23)^. Therefore, it is necessary to continue carrying out other studies to clarify this relationship. So, it is suggested that, in the analysis of the association of this variable, aspects such as professional competence, training and emotional support received by health personnel be incorporated.

One of the predictors that had a moderate psychological impact in this study was self-perceived mental health. Likewise, a study carried out in Brazil showed that a psychiatric history is associated with post-traumatic stress, anxiety and depression, caused by the COVID-19 pandemic^(19)^. In addition, another study reports that those who determine a poor health status have a greater psycho-emotional impact^(10)^. This can be understood from the perspective that people are relatively efficient when it comes to introspectively assessing the mental health problems that may afflict them. This finding is particularly useful because, in situations where it is not possible to access validated questionnaires, self-perception of mental health allows an approximation to a person’s psychological problems.

Finally, when exploring the perception of media information, the analysis reported that it is only associated with depression. However, a study carried out in Brazil points out that the intensity of exposure to news of the pandemic is related to the psycho-emotional impact^(19)^. Another study carried out in the Philippines indicates that satisfaction with the amount of health, information available on COVID-19, is associated with less depression, anxiety and stress^(10)^.

### Contributions to the area of nursing, policy or public health

The findings of this study have implications for the prevention of psycho-emotional problems in the nursing team that performs care activities, as it was shown that the self-perception of their mental health and the type of religion they profess are associated with the psycho-emotional impact. In addition, it is reported that an unsafe work environment characterized by the existence of mistreatment by patients generates greater depression, anxiety and stress in nurses.

### Limitations of the study

Among the limitations that the study presented is the data collection method, as it is an online questionnaire, the results may be influenced by a social desirability bias. Furthermore, having opted for a non-probabilistic sampling does not allow the generalization of the results. Regarding the experience of abuse, the frequency or form of abuse was not investigated, an aspect that could influence the association with the psycho-emotional impact.

## CONCLUSIONS

In this study, it is evident that the psycho-emotional impact of COVID-19 was characterized by the fact that half of the nurses had some degree of anxiety, approximately one third of depression and two out of 10, some degree of stress. Predictive models indicate that participants who profess the evangelical religion, agnostics, those who self-perceived as regular or poor mental health and those who have already suffered some experience of abuse by patients are more likely to present stress, anxiety and depression. Likewise, those professionals with more than 15 years of professional experience tend to present greater stress and anxiety. On the other hand, male employees and those who perceive media information as inadequate may exhibit depression.

## Data Availability

https://doi.org/10.48331/scielodata.FZQR77
